# Navigating the landscape of the unfolded protein response in CD8^+^ T cells

**DOI:** 10.3389/fimmu.2024.1427859

**Published:** 2024-07-04

**Authors:** Keith Alan Nair, Bei Liu

**Affiliations:** ^1^ Division of Hematology, Department of Internal Medicine, The Ohio State University Comprehensive Cancer Center, Columbus, OH, United States; ^2^ The Pelotonia Institute for Immuno-Oncology, The Ohio State University Comprehensive Cancer Center, Columbus, OH, United States

**Keywords:** endoplasmic reticulum stress, unfolded protein response, IRE1, PERK, ATF6, CD8 T cell

## Abstract

Endoplasmic reticulum stress occurs due to large amounts of misfolded proteins, hypoxia, nutrient deprivation, and more. The unfolded protein is a complex intracellular signaling network designed to operate under this stress. Composed of three individual arms, inositol-requiring enzyme 1, protein kinase RNA-like ER kinase, and activating transcription factor-6, the unfolded protein response looks to resolve stress and return to proteostasis. The CD8^+^ T cell is a critical cell type for the adaptive immune system. The unfolded protein response has been shown to have a wide-ranging spectrum of effects on CD8^+^ T cells. CD8^+^ T cells undergo cellular stress during activation and due to environmental insults. However, the magnitude of the effects this response has on CD8^+^ T cells is still understudied. Thus, studying these pathways is important to unraveling the inner machinations of these powerful cells. In this review, we will highlight the recent literature in this field, summarize the three pathways of the unfolded protein response, and discuss their roles in CD8^+^ T cell biology and functionality.

## 1 Introduction

The endoplasmic reticulum (ER) is a complex and robust cellular organelle that specializes in the synthesis and folding of proteins, the biogenesis of lipids, and calcium metabolism (1). The ER is composed of two different parts: the rough, sheet-like ER and the smooth, tubule ER (2). The rough ER is characterized by the large amounts of ribosomes on the surface of its sheets, while the smooth ER is characterized by the non-appearance of ribosomes (3). While the smooth ER is known for lipid production and calcium storage, the rough ER is the primary location of protein synthesis and modification (1, 3). The ER is also the regulator of the translation of secretory proteins as well, which commonly occurs on the ER membrane (4, 5), whereafter they translocate into the ER lumen and are properly folded (6, 7).

Proteostasis, the maintenance of the balance of proteins in a cell, is an integral component of maintaining the health of the cell (8). Proteostasis is maintained by protein chaperones, needed for conformational changes in developing proteins, and both the ubiquitin-proteasome and lysosome-autophagy systems, necessary for degrading un- or misfolded proteins (9). However, an accumulation of misfolded proteins can occur, leading to ER stress and thus activating the unfolded protein response (UPR) (10). This can be caused by several factors, such as hypoxia, nutrient deprivation, reduced glycosylation, ineffective degradation of proteins, errors in post-translation modifications, lipid bilayer alterations, and calcium level shifts (11, 12).

**Figure 1:**
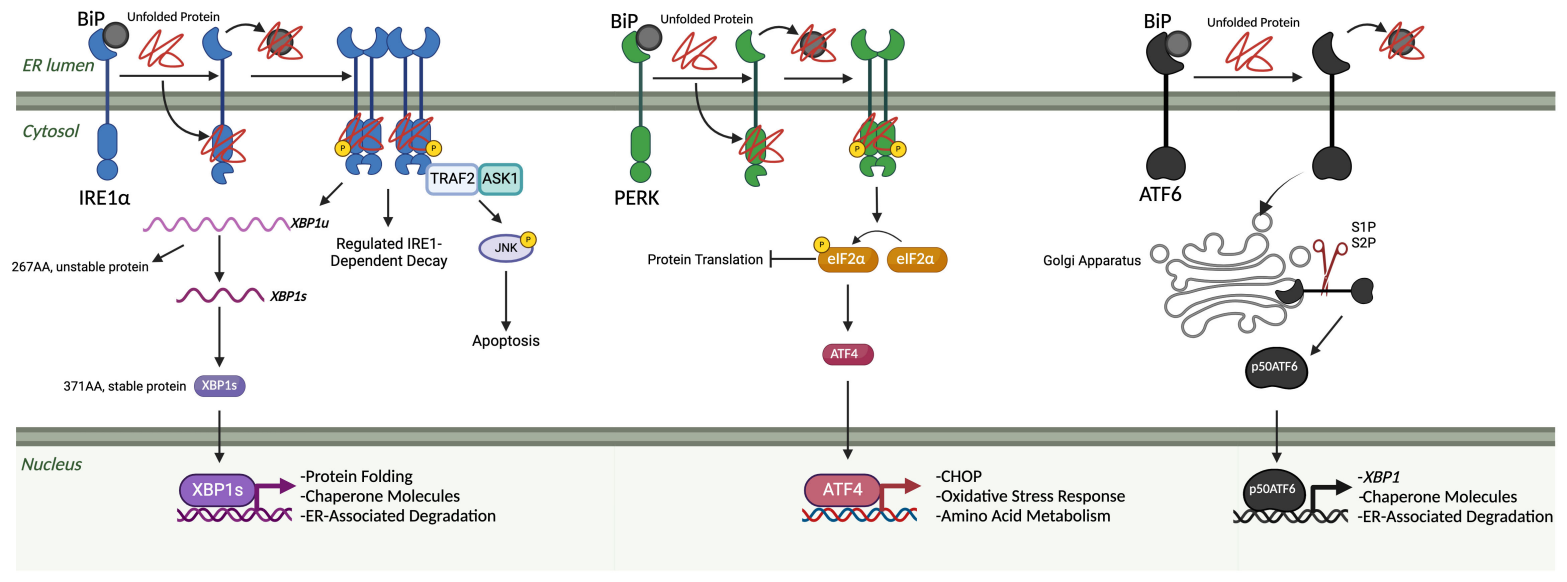
The unfolded protein response. The three arms of the UPR are IRE1α, PERK, and ATF6. Under homeostatic, non-stressed conditions, these three arms are held in an inactivated state by BiP. Once misfolded proteins accumulate in the ER lumen, BiP binds to those and releases them from the three arms, leading to their subsequent activation. Once free, IRE1α begins to oligomerize and auto-phosphorylate, leading to the start of its endoribonuclease activity. Two primary outcomes associated with IRE1α endoribonuclease activity: splicing of XBP1 mRNA into the XBP1s transcription factor and regulated RIDD. XBP1s translocates to the nucleus, where it upregulates genes related to protein folding, chaperone molecules, and ER-associated degradation elements. IRE1α also associates with a TNF-receptor associated factor 2 (TRAF2) – apoptosis signal-regulating kinase 1 (ASK1) complex, which results in the phosphorylation of c-Jun N-terminal kinase (JNK) and downstream apoptosis. When PERK is unbound, it activates in a similar oligomerization and auto-phosphorylation manner as IRE1. Once activated, it acts primarily through the phosphorylation of eIF2α. Then, eIF2α can lead to both protein translation inhibition and the favoring of the translation of ATF4. ATF4 then translocates to the nucleus and can upregulate CHOP, oxidative stress response elements, and amino acid metabolism elements. Once unbound from BiP, ATF6 will travel to the Golgi apparatus, where it is cleaved by site-1 and site-2 proteases. Once cleaved into a 50-kDa protein, p50ATF6 translocates to the nucleus, which can upregulate *XBP1*, chaperone molecules, and ER-associated degradation elements. (Created via BioRender).

The UPR was initially hypothesized in a work published by Kozutsumi et al. in 1988, in which they deduced the presence of two major chaperone proteins induced by ER stress: GRP78 and GRP94 (13). It was eventually revealed that when unfolded proteins reach a certain concentration in the lumen of the ER, aggregation is considered significant, leading to imbalance and activation of the UPR (14, 15). To detect shifts in proteostasis, the UPR uses three unique sensors: inositol-requiring protein 1 (IRE1), protein kinase RNA-like ER kinase (PERK), and activating transcription factor-6 (ATF6). The UPR has three general methods of clearance using these sensors and their downstream functions: depletion, upregulation, and apoptosis (16) (**Figure 1**).

CD8^+^ T cells are one of the most extensively studied immune cell subsets, often being compared to soldiers, as they are the body’s targeted weapon against insults (17). These cells are primed through three distinct mechanisms: 1) T cell receptor being presented with an antigen by major histocompatibility complex-I (MHC-I) from an antigen-presenting cell; 2) co-stimulation with various ligands; and 3) cytokines supporting the activation and differentiation (18). Once primed, they traffic from the lymph nodes to the site of insult, wherein they gain their effector program upon re-engagement of the TCR-MHC complex and co-stimulation molecules (19, 20). However, the role of the UPR in CD8^+^ T cells has been shown to have a wide-ranging spectrum of effects on these processes. In this review, we will further explore the functions of these pathways and their roles in CD8^+^ T cell biology and functionality.

## 2 The IRE1 pathways

### 2.1 Overview of the IRE1/XBP1 pathway

IRE1 was discovered as a transmembrane kinase in *Saccharomyces cerevisiae* in 1992 and speculated to play a role in signal transduction (21). It wasn’t until one year later that its role was deduced when it was shown that IRE1 is able to transduce signals from the ER to the nucleus (22, 23). Then, IRE1 was characterized as a serine-threonine protein kinase initiated by unfolded protein accumulation, where it then oligomerizes and is activated by trans-autophosphorylation (24, 25). Also, IRE1 has endoribonuclease activity that cleaves the *HAC1* mRNA into a transcription factor (TF) that translocates to the nucleus and controls the UPR. Previously, this was all shown in yeast, but a breakthrough came when a yeast IRE1 homolog was found in mammalian cells and had similar trans-autophosphorylation and endoribonuclease activity. It also splices yeast *HAC1*, revealing a homologous protein may exist in mammalian cells (26). The same group found that the IRE1 endoribonuclease activity is autoregulatory to its own mRNA and theorized the presence of two human isoforms of IRE1 (27). These two forms of human IRE1 were identified and characterized as IRE1α and IRE1β, each with distinct functions. IRE1α was shown to be ER stress-related, while IRE1β was found to cleave 28s ribosomal RNA, causing translational repression (28). The disparities in function were later attributed to structural differences in the RNase domain of the isoforms (29).

The primary splicing target of IRE1α in mammals was later discovered to be X-box binding protein 1 (XBP1) (30, 31). XBP1 was first identified in 1996 as a basic region leucine zipper (bZIP) TF (32) that acts on several transcriptional elements, later identified as the unfolded protein response element (UPRE) and the endoplasmic reticulum stress element (ERSE) I and II (31, 33). XBP1 binds at CCACG sequences in the ERSE regions and the palindrome sequence (CGTGC) in the UPRE regions (30, 32). XBP1 was shown to be two separate protein products named based on their form: an unstable, unspliced XBP1u or a stable, spliced XBP1s (34). IRE1α excises a 26 nucleotide intron from the *XBP1* mRNA that is then re-ligated and results in a frameshift mutation, extending the open reading frame and becoming a stable 376 amino acid protein (34, 35). The RNA ligase RtcB is responsible for ligating the cleaved *XBP1* mRNA together after IRE1α excision (36, 37). RtcB is regulated via phosphorylation by ABL1 and PTP1B (38, 39). XBP1s has several functions, such as upregulating the ER degradation-enhancing α-mannosidase-like protein (EDEM), which is critical for ER-associated degradation (ERAD), as well as several other proteins involved in relieving ER stress, such as chaperones (40, 41). It also plays an important role in glucose and lipid metabolism, inflammatory pathways, collagen secretion, pro-insulin synthesis in β-cells, and several other processes (41–46).

#### 2.1.1 Activation of yeast IRE1

The method of activation of IRE1 has undergone much debate, but three models have been proposed: a direct or indirect model and one in which it’s a synergistic combination. In 2000, it was shown that a protein called Kar2p (also known as GRP78 or BiP) is bound to the ER lumen region of IRE1, interacts and binds with unfolded proteins, and is released, causing the sequence of activating events for IRE1 in yeast (47), which led to the initial hypothesis that the UPR is via an indirect model in which BiP binding to unfolded proteins leads to its disassociation with the monomeric IRE1, thus activating the downstream IRE1 pathways. There was a challenge when Kimata et al. showed that BiP binding to IRE1 is not required to prevent constitutive activation of IRE1, demonstrating that BiP may play a role as an adjustor of the UPR (48). Crystallography analysis of IRE1 revealed the structure of the luminal domain (LD) as containing a deep groove similar to MHC, suggesting that activation may be dependent on that domain being occupied by unfolded proteins, leading to a proposed direct activation model (49). It was later hypothesized that BiP disassociation leads to monomeric IRE1 binding to unfolded proteins via the LD, thus inducing a conformational change, oligomerization, and activation. Once stress is resolved, BiP can rebind to IRE1, leading to deactivation via monomerization (50). Furthermore, studies showed that the LD of yeast IRE1 directly bound to unfolded proteins caused oligomerization and activation of the UPR functions (51).

#### 2.1.2 Activation of mammalian IRE1

BiP was discovered at a similar time in mammalian cells as in yeast and hypothesized to directly repress IRE1 functions (52). The indirect model was proposed in which unfolded proteins sequester BiP away from IRE1, which allows the hydrophobic regions to dimerize and trans-autophosphorylation to occur (53). The activation of IRE1 was later determined to be reliant on at least four IRE1 monomers forming an oligomer (54), which was confirmed later when an IRE1α mutant was generated in mammalian cells with a low-affinity BiP binding site, showing that in the absence of BiP binding, IRE1α is activated under homeostatic conditions (55). Although it was proposed that BiP is tapered from the LD of IRE1 during ER stress (16), there was still the question of the sensitivity of the UPR. Importantly, even under low amounts of ER stress, the pathway can still be activated (49). One study introduced a new aspect of BiP that can explain this phenomenon. There is a non-canonical interaction between IRE1 LD and BiP’s ATPase domain that retains it in the monomeric state that is detached once unfolded protein binds to the substrate binding domain of BiP, indicating an allosteric regulatory role (56). This was further explored by the discovery of ER-localized J-protein 4 (ERdj4) and its role in drawing BiP towards dimerized IRE1 (57). BiP has a low affinity for the LD of IRE1. Thus, the complex is conserved through an ATP-mediated process that involves a release via nucleotide exchange, such as with a protein like GRP170, and then reunification with the help of a J-protein like ERdj4 (57, 58). ERdj4 also plays an active role in the suppression of IRE1. ERdj4 binds to IRE1 dimers via the C-terminal targeting domain and recruits BiP to break apart the dimers via ATP hydrolysis with its J domain, in which ERdj4 is then released (57). Karagöz et al. showed that mammalian IRE1 can be activated through unfolded proteins binding to the LD of IRE1 and hypothesize that BiP functions as a tuning mechanism. Importantly, they also show that BiP and IRE1 LD prefer different subsets of peptides. BiP prefers serine and threonine residues, and IRE1 LD prefers prolines and histidines. The group hypothesizes that unfolded protein binding in the LD leads to rearrangements that allow stabilization of the oligomeric form of IRE1, then allows trans-autophosphorylation and the activation of downstream functions (59). Work done in this field is still ongoing, and a clear consensus has not been reached yet. Considering the data, the true model seems to lie in the synergized combination. BiP appears to disassociate with IRE1 once unfolded proteins interact with it, which induces a conformational change in the LD of IRE1 that enables unfolded protein binding. This allows for the domains of IRE1 to interact and oligomerize, trans-autophosphorylate, and thus become active. When ER stress is resolved, IRE1 begins to disassociate from its oligomeric form, and ERdj4 binds to it, which then recruits BiP and leads to an ATP hydrolysis reaction that binds BiP to the LD of IRE1 and releases ERdj4, causing IRE1 to become monomeric and inactivated.

#### 2.1.3 Alternative activation pathways

The phosphorylation of IRE1α is also vital, with three regions needing to be phosphorylated: the activation loop, the linker region, and the RNase domain (60). The activation loop phosphorylation is necessary for the enhancement of the splicing of XBP1 (60) and the locking of IRE1α into an active state for oligomerization (61). Alternatively, several non-canonical methods can activate IRE1. For example, membrane aberrancy (62) and changes in lipid composition (63) activate the IRE1 pathway. Also, IRE1 is sensitive to the thickness and the amount of lipid packing in the ER membrane through an amphipathic helix (64, 65). HSP47, a collagen chaperone, was discovered to bind to the IRE1α LD and reduce BiP binding, engaging the UPR (66), which provides a link to the UPR and collagen synthesis and trafficking, as TANGO1, a protein that assists in the secretion of collagen, is upregulated by XBP1s (44).

### 2.2 Non-canonical IRE1 functions

IRE1 has various physiological functions that primarily center around regulating cell death and re-establishing homeostasis after ER stress. One of the first functions described was the ability of IRE1 to regulate caspase-12 during ER stress. If ER stress reaches a critical level, an IRE1α-TRAF2-ASK1 complex can induce apoptosis through the JNK signaling pathway that causes caspase activation (67–69). Pro-apoptotic proteins Bax and Bak also directly interact with IRE1, wherein Bax and Bak appear to be necessary for the lessening of ER stress in pancreatic islet cells, and in a double knockout of these two proteins, IRE1 and XBP1s were both significantly upregulated (70). Sustained expression of IRE1α can also destabilize miRNA-17, which leads to increased thioredoxin-interacting protein (TXNIP) levels. When this occurs, the NLRP3 inflammasome is activated, leading to downstream cleavage of caspase-1, IL-1β secretion, and cell death (71, 72). An important discovery was made in Drosophila when it was discovered that, besides cleaving *XBP1*, the IRE1α endoribonuclease domain is also vital for cleaving and causing degradation of mRNAs for secretory proteins in a process called regulated IRE1-dependent decay (RIDD) (73). This process was later shown to be conserved in mammalian studies (74), with one study specifically showing that RIDD appears to be activated during more severe ER stress, whereas *XBP1* splicing occurs during low-level stress (75). Although the consensus sequence was CUGCAG and a stem-loop structure for canonical cleavage targets like *XBP1* and *CD59* (76), it was unknown what this sequence was for RIDD targets. In 2021, Le Thomas et al. described a promiscuous form of RIDD, titled RIDDLE or RIDD lacking endomotif. RIDD requires dimerization of IRE1α and is shown by this group to recognize and cleave the CNGCAGN sequence and stem-loop structure, whereas RIDDLE requires a higher-order phospho-oligomerization of IRE1α and is promiscuous. Specifically, it can degrade non-optimal or non-existent stem-loop endomotifs and consensus RIDD substrates (77). The ability of IRE1 to perform RIDD is a direct effector function for it to relieve ER stress by lowering the amount of transcripts present. RIDD also has other unique roles, such as cleaving a group of miRNAs that repress the pro-apoptotic protein caspase-2 (78) and cleaving miRNAs that suppress angiopoietin, leading to better wound healing in a diabetic model (79). IRE1α is vital in the placenta; in a knockout model, mice died at the embryonic stage for reasons that appear to be linked to VEGF-A (80) and β-cell proinsulin folding (81). Related to the topic of β-cell proinsulin, it has also been reported that IRE1α splicing of *XBP1* mRNA is required for accelerating the synthesis of proinsulin in response to elevated blood glucose levels (45). Acosta-Alvear later showed that IRE1 interacts with a signal recognition particle on the surface of ribosomes, arriving at the ER translocon in two unique ways: preemptive mode and surveillance mode. In the preemptive mode, IRE1 is able to degrade the mRNA before it enters the ER, and in the surveillance mode, it can degrade proteins that have folding issues as it is translated and pushed through the translocon (82). Targeting this pathway therapeutically has led to positive results in diseases such as cancers, renal failure, and diabetes (**Table 1**).

**Table 1:** Potential therapeutic compounds for the treatment of UPR-related disorders.

Compound	Mechanism of Action	Diseases Tested	Sources
4µ8C	IRE1α RNase Inhibitor	Colon Cancer	(83, 84)
AMG18 (KIRA8)	IRE1α RNase Inhibitor	MM, Non-Obese Diabetes	(85–87)
B-I09	IRE1α RNase Inhibitor	CLL	(88)
Bortezomib	Proteasome Inhibitor (Activates UPR)	MM	(89)
CAY10566	Increases Expression of BiP, CHOP, XBP1s	GBM	(90, 91)
Compound 147	ATF6 Activation	Ischemia/Reperfusion Injury	(92)
Dibenzoylmethane	Reduces ATF4 and CHOP Levels, Increases BiP	Dementia	(93, 94)
GSK2606414	PERK Inhibitor	Neuroblastoma, Neurodegeneration	(95–97)
GSK2656157	PERK Inhibitor	Pancreatic Tumor, MM	(98, 99)
Guanabenz	Inhibits eIF2α Dephosphorylation	Hypertension, ALS	(100–102)
HA15	BiP Inhibitor	Lung Cancer, MM	(103–105)
IKM5	BiP Inhibitor	Breast Cancer	(106)
ISRIB	Blocks Effects of eIF2α Phosphorylation	ALS, Prion Diseases	(107–110)
KIRA6	IRE1α RNase Inhibitor	Diabetes, Retinal Degeneration	(111)
KP1339	BiP Inhibitor	Colon Cancer	(112, 113)
MKC3946	IRE1α RNase Inhibitor	MM	(114)
MKC8866	IRE1α RNase Inhibitor	Breast Cancer, GBM	(115, 116)
OSU-03012	BiP Inhibitor	Osteosarcoma	(117, 118)
Salubrinal	Inhibitor of eIF2α Dephosphorylation	Breast Cancer, Iron-Induced Insulin Resistance	(119, 120)
Sephin1	Inhibitor of eIF2α Dephosphorylation	MS	(121)
STF-083010	IRE1α RNase Inhibitor	ARF, MM, Breast Cancer	(122–124)
Trazodone	Reduces ATF4 and CHOP Levels	Dementia, Prion Diseases	(94, 125)

ALS, Amyotrophic Lateral Sclerosis; ARF, Acute Renal Failure; CLL, Chronic Lymphocytic Leukemia; GBM, Glioblastoma; MM, Multiple Myeloma; MS, Multiple Sclerosis.

### 2.3 IRE1/XBP1s pathway in CD8^+^ T cells

Initially, using an IRE1-GFP reporter model, a previous study showed that IRE1 was upregulated during the double-positive thymic development stage yet only remained upregulated in CD8 single-positive T cells in both the thymus and spleen (126). However, the role of IRE1 in T cell development is still an area of active research, as the loss of both IRE1 and XBP1 in T cells doesn’t affect their development or frequency (127, 128). IRE1 may play a role in regulating TCR arrangement, although more research is needed in this area. Interestingly, one group showed that IL-2 can induce *XBP1u* transcripts, while TCR ligation initiates IRE1α activation to cleave *XBP1u* into *XBP1s* (129). In mice infected with ovalbumin-expressing *Listeria monocytogenes* (LM-OVA), both *ERN1* (IRE1) and *ATF6* mRNA were shown to increase 12 hours post-infection via a transcriptome analysis (130). It was later revealed that TCR ligation activates protein kinase C, which leads to the induction of the UPR, specifically BiP levels increasing. Interestingly, it was also shown that the phorbol 12-myristate 13-acetate (PMA) and ionomycin, common CD8^+^ T cell activating compounds *in vitro*, activate the UPR also due to protein kinase C activation (131). This indicates a regulatory system between calcium signaling, T cell activation, and the UPR that further research is required to understand fully. Similarly, store-operated calcium entry (SOCE) is a critical step for T cell activation (132) and is promoted via IRE1 and STIM1 interactions to increase ER and plasma membrane contact sites (133).

**Figure 2:**
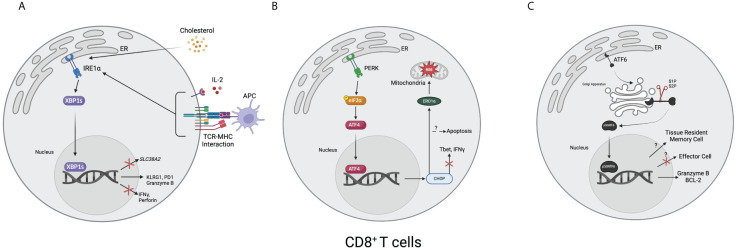
Overview of the unfolded protein response in CD8^+^ T cells. **(A)** T cell receptors and MHC combined with interleukin-2 result in the upregulation of XBP1s. Cholesterol, such as from the tumor microenvironment, can also lead to upregulating XBP1s. Once upregulated, XBP1s can increase the expression of KLRG1, PD1, and granzyme B and decrease the expression of IFNγ, perforin, and *SLC38A2*, a gene encoding for a glutamine transporter. **(B)** After activating, ATF4 translocates to the nucleus and upregulates CHOP, resulting in decreased Tbet and IFNγ. It can also result in apoptosis. Downstream, CHOP increases endoplasmic reticulum oxidoreductase 1α (ERO1α), leading to an accumulation of mitochondrial reactive oxygen species (ROS). **(C)** After the 50-kDa portion of activating transcription factor 6 (p50ATF6) translocates to the nucleus, it has hypothesized effects on enforcing tissue-resident memory cell formation and downregulating effector cell formation. It has also been shown to bind to and upregulate granzyme B and B-cell lymphoma 2 (BCL-2). (Created via BioRender).

In both LM-OVA and LCMV models, XBP1s supports CD8+ T cell differentiation into end-stage effector cells via enhanced KLRG1 expression, although it is dispensable for the differentiation state (129). IRE1α inhibition in CD8^+^ T cells was also shown to result in immunosuppression due to lower effector functionality and memory commitment, thus the rejection of the transplant was lessened (134). While studying hereditary sensory and autonomic neuropathy type I (HSAN-I), one group found that serine palmitoyltransferase long chain base subunit 2 (SPTLC2) dysfunction results in increased mTORC1 activation and ER stress, specifically XBP1s and CHOP upregulation, thus resulting in CD8^+^ T cell apoptosis (135). Human CD8^+^ T cells isolated from ovarian cancer ascites also showed increased XBP1s expression compared to healthy donor cells, and the knockout of XBP1s in CD8^+^ T cells in a murine model showed higher perforin and IFN-γ production (136). Another example of the role of XBP1s in CD8^+^ T cells was revealed when a research group showed that increased cholesterol in the TME disrupts lipid metabolism, leading to ER stress, higher XBP1s and immune checkpoint expression, and fewer effector molecules (137). Recently, a group published that in a comparison between healthy donor and multiple myeloma CD8^+^ T cells, there is increased XBP1s that they show bind to and decrease *SLC38A2*, a gene encoding for the glutamine transporter SNAT2 (138). Within 24 hours of antigen activation in a murine model of LCMV *in vitro* (139), it was found that protein synthesis tripled in CD8^+^ T cells, indicating that there may be a need for a protein regulation system such as the UPR. An incomplete image of how IRE1 and XBP1s regulate CD8^+^ T cells is still present, and further research will need to focus on the mechanisms of how these molecules regulate CD8^+^ T cells. Currently, it appears that this pathway regulates CD8^+^ T cells via multiple mechanisms: managing protein burden, calcium signaling and activation, metabolism, and exhaustion. How, or if, these pathways intersect is unknown, although it is plausible that the activation of CD8^+^ T cells upregulates the UPR via calcium signaling, which results in the downregulation of metabolite transporters, like SNAT2, and thus T cell exhaustion (**Figure 2A**).

## 3 The PERK pathway

### 3.1 Overview of the PERK pathway

PERK was identified as a type I transmembrane-ER-resident protein that is similar to IRE1. It contains an ER-stress-sensing luminal domain and a cytoplasmic region that is structurally similar to the eukaryotic initiation factor 2 (eIF2α) kinases (140). Once activated by ER stress, PERK autophosphorylates and begins its protein kinase activity by phosphorylating eIF2α on serine 51, which inhibits the translation of select mRNA (139, 140). Phosphorylation of Thr980 of PERK stabilizes the activation loop and the C-terminal helix αG, which was shown to be important for preparing it for binding to eIF2α (141, 142). The conformational change of PERK was also shown to be vital for increasing the binding affinity of the kinase loop to eIF2α (143). After phosphorylation, eIF2α was discovered to increase the translation of activating transcription factor 4 (ATF4), which induces the protein C/EBP-homologous protein (CHOP) (144). ATF4 plays an important role in promoting resistance to oxidative stress, mediating amino acid metabolism, and regulating angiogenesis (144–146). The activation of PERK was later discovered to be linked to BiP disassociating from it, in which, after disassociation, PERK will oligomerize, trans-autophosphorylate, and then activate similarly to IRE1 (52). It was also hypothesized that PERK can bind unfolded proteins in its luminal domain through an MHC-like groove, which will cause the molecules to line up and form dimers and oligomers (52, 147). PERK activation can result in the disassociation of Nrf2/Keap1 complexes, which leads to Nrf2 translocating to the nucleus and increasing cell survival pathways in an eIF2α phosphorylation-independent manner (148).

### 3.2 PERK functions

In an early experiment, PERK was knocked out in murine cells, and it was shown that there was a decrease in the inhibition of protein synthesis, phosphorylation of eIF2α, and cell survival when exposed to ER-stress-inducing agents (149). It has also been shown that cells can be caught in G1 arrest in the cell cycle when PERK is activated due to eIF2α phosphorylation and the loss of cyclin D1 (150). PERK plays an important role in ER-mitochondria contact sites and mitochondrial-associated ER membranes (MAMs) by transducing crosstalk that can induce reactive oxygen species cell death and regulate Ca^2+^ stores (151). Then, Muñoz et al. discovered that the key GTPase in the MAM site is mitofusin 2 (Mfn2), which regulates the transfer of Ca^2+^ between the ER and mitochondria. Mfn2 is a regulator of PERK that can keep PERK inactive under homeostatic conditions and can also detect cellular stress to coordinate the activation of PERK. They also showed that PERK was able to control the morphology and function of mitochondria, as well as oxidative stress (152). Although it was previously believed that Ca^2+^ perturbations can activate PERK (16, 151), the link was not fully clear until van Vliet et al. discovered that Filamin A was the protein that interacts with dimerized PERK once Ca^2+^ levels rise in the cytosol to drive actin remodeling. These events lead to an increase in stromal interaction molecule 1 (STIM1), which increases the contact sites between the ER and plasma membrane to refill the ER Ca^2+^ stock (153). Similar to IRE1, PERK has also been implicated in playing an important role in β-cell pathways; when PERK is knocked out in mice, it leads to Wolcott-Rallison syndrome, a severe infantile diabetes mellitus (154). By repressing the levels of the NF-κB inhibitor IκBα, eIF2α plays an important role in regulating the wide-ranging effects of NF-κB in the cells, although a physiological link has not yet been shown (155). This phenomenon has been shown to occur through ultraviolet light exposure as well (156). eIF2α phosphorylation is also necessary for β-cell insulin trafficking and upregulation of various transcription factors; without it, it can lead to β-cell dysfunction and diabetes mellitus (157, 158). Additionally, the PERK pathway has been implicated in the field of neurology, including improving cognitive memory (107) and controlling cortical neurogenesis (159).

### 3.3 PERK pathway regulatory mechanisms

While the binding of PERK with BiP is the initial regulatory mechanism in the pathway, there are several others as well. One of the first mechanisms discovered that regulated the PERK pathway was uncovered by Novoa et al. and revolved around a protein called growth arrest and DNA damage-inducible 34 (GADD34) associating with protein phosphatase 1 (PP1c), which in turn reduced the levels of eIF2α phosphorylation. This results in lowered amounts of ATF4 and CHOP, lessening the effects of the PERK pathway (160). The constitutive repressor of eIF2α phosphorylation (CReP) was later discovered and was also shown to associate with PP1c, leading to dephosphorylated eIF2α and a lessened stress response (161). One group discovered that, despite their low amino acid similarity, GADD34 and CReP have a common function: both are vital for regulating eIF2α phosphorylation and the loss of both results in embryonic lethality (162). P58^IPK^ is upregulated by ER stress and can inhibit PERK signaling and phosphorylation, which will reduce the activation of the pathway, resulting in a negative feedback loop (163, 164). Protein disulfide isomerase A6 (PDIA6) attenuates the response of the UPR by inhibiting the luminal domain of both IRE1 and PERK (165). An ER-localized transmembrane protein called transducin beta-like 2 (TBL2) was discovered to associate with activated PERK and to positively regulate the downstream post-transcriptional processing of ATF4 (166). Related to the aforementioned role of PERK in calcium homeostasis, calcineurin (CN), a Ca^2+^ and calmodulin-dependent phosphatase, binds to PERK and can increase autophosphorylation and thus the activity of the UPR, resulting in CN playing the role of a positive regulator of ER stress (167). Rheb, a protein involved in the mTORC1 protein synthesis pathway, has also been shown to interact with PERK, thus increasing the efficiency of PERK phosphorylation onto eIF2α (168). Interestingly, nitric oxide was discovered to affect PERK via S-nitrosylation by Nakato et al. S-nitrosylation of PERK results in higher phosphorylation of eIF2α, thus increasing the pro-apoptotic pathways (169).

CHOP, the prominent effector protein expressed by the PERK pathway, was first discovered by a group looking for DNA-damage-inducible genes. Instead, they found only one that was upregulated during both heat shock stress and DNA damage as well as growth-attenuating conditions: growth arrest and DNA damage-inducible 153 (GADD-153) (170, 171). Previously only shown in hamster cells, the homologous protein was later identified in murine cells as C/EBP-homologous protein 10 (CHOP-10), a protein that forms heterodimers with C/EBP-like proteins that inhibit their binding to their respective DNA enhancer elements (172). It was then shown that this heterodimer complex can also be phosphorylated, as well as activate genes known as downstream of CHOP (DOCs) (173). The first link to the UPR with CHOP was uncovered when, after cells were exposed to stress-causing conditions, CHOP was upregulated quickly yet was attenuated by overexpression of BiP (174). ATF4 was then discovered as the necessary TF that upregulates CHOP during ER stress, linking the PERK pathway and CHOP (175). Interestingly, in a positive feedback loop, CHOP is also able to interact with ATF4, which leads to increased protein synthesis as well as oxidative stress and cell death (176). The primary role of CHOP, apoptosis (177), was originally shown to be due to CHOP decreasing the levels of B-cell lymphoma 2 (BCL-2), causing cells to die (178). This was later shown to be unlikely; rather, the BCL-2 family member protein called Bim is the increased pro-apoptotic protein due to transcriptional induction from CHOP (179). Death receptor 5 (DR5), a protein involved in caspase 8-mediated apoptosis, was discovered to be upregulated by CHOP during ER stress by Lu et al. In an interesting interplay between UPR pathways, IRE1 cleaves the DR5 mRNA, leading to no apoptosis, but if ER stress continues, IRE1 levels begin to wane while PERK and CHOP remain consistent, leading to enhanced DR5 expression and apoptosis (180). Marciniak et al. discovered that CHOP activates GADD34, resulting in a negative feedback loop for the PERK pathway. They were also able to show that CHOP can directly activate endoplasmic reticulum oxidoreductase 1α (ERO1α), leading to oxidizing conditions in the ER and thus apoptosis (181). Similar to PERK, CHOP is vital to controlling β-cell failure in diabetes; when knocked out in a murine model, the mice had higher glycemic control and lower oxidative damage (182). Inhibitors of the PERK pathway have been used for diseases such as neuroblastomas, multiple myeloma, and multiple sclerosis (**Table 1**).

### 3.4 PERK pathway in CD8^+^ T cells

CHOP was shown to be induced when GTPase of the immunity-associated nucleotide-binding protein 5 (Gimap5) was lost, resulting in apoptosis of rat CD8^+^ T cells (183). Cao et al. discovered that CHOP was upregulated in CD8^+^ T cells from tumor-bearing mice, which resulted in negative regulation of Tbet. Deletion of CHOP from CD8^+^ T cells significantly reduces tumor burden via increased IFN-γ and Tbet expression (184). Another study by Hurst et al. zoomed out from CHOP, instead using both a PERK knockout and a PERK inhibitor to show that tumor burden, mitochondrial exhaustion, and reactive oxygen species are all decreased. ERO1α, a protein upregulated by CHOP and involved in protein folding through oxidation reactions, was shown to be a large contributor to their phenotype via the accumulation of reactive oxygen species and subsequent damage to the mitochondria. They also used combination therapy with anti-PD1 and the PERK inhibitor, showing significantly increased tumor clearance and survival (185). KDEL receptor 1 (KDELR1) was identified as an associate of protein phosphatase 1 (PP1). Thus, without KDELR1, PP1 does not perform its phosphatase activity on eIF2α, resulting in increased CHOP in CD8^+^ T cells and apoptosis (186). Solanki et al. showed that ribosomal protein L22 (RPL22) resulted in significant ER stress if lost, leading to the induction of the arresting protein p53. In rescue experiments, they found that the knockdown of PERK specifically reduces p53 induction and rescues the T cell, thus showing a link between the cell cycle and the UPR (187). Cytoplasmic polyadenylation element-binding protein 4 (CPEB4) regulates the UPR in CD8^+^ T cells. When CPEB4 is deleted, there is weaker anti-tumor immunity and significantly increased CHOP expression (188). Stimulators of IFN genes (STING) regulate ER stress and T cell survival primarily through calcium homeostasis (189) and CHOP (190). While the knowledge of how the PERK pathway regulates CD8^+^ T cells is still incomplete, the data indicates that PERK upregulation, and subsequent CHOP upregulation, are both negative regulators of T cell immunity via oxidative stress and the downregulation of effector transcription factors and cytokines (**Figure 2B**).

## 4 The ATF6 pathway

### 4.1 Overview of the ATF6 pathway

ATF6 was initially discovered in 1998 as a basic leucine zipper (bZIP) TF that binds ER stress response elements (ERSE) to resolve ER stress (191). ATF6 binds to the consensus CCAAT-N_9_-CCACG sequence, specifically binding to the CCACG sequence when the CCAAT sequence is bound by the NF-Y TF upstream (192). The TF YY-1 (193) and TBP (194) were also shown to be factors that can increase the upregulation of ERSE transcription via interactions with ATF6. p90ATF6 exists as a transmembrane protein in the ER, but it is proteolyzed during ER stress, leading to activated p50ATF6 that is translocated to the nucleus to upregulate the ERSE, such as BiP (194, 195). While the activation mechanism was unknown for some time, it was discovered that BiP plays an inhibitory role, wherein it binds to Golgi localization sites on ATF6 and unbinds during ER stress, allowing it to translocate (196). Shortly after the initial discovery, ATF6’s cleavage was shown to be mediated by site 1 and site 2 proteases (S1P and S2P) (197). The Golgi apparatus was discovered to be the site of this cleavage, with the LZIP protein being an important player in translocating ATF6 after recognizing ER stress via the luminal domain, where it is then cleaved and shuttled to the nucleus (198). The mechanism of translocation from ER to the Golgi was later shown to be mediated by COPII vesicles once ATF6 is unbound from BiP (199). ATF6, similar to IRE1, was also shown to exist in two distinct forms: ATF6α and ATF6β (200). Although it was initially shown that knocking out both forms was not vital to cell survival (41), it was disproven in a murine model when knockout of the two isoforms was shown to be embryonic lethal. The same study also showed that ATF6a is the isoform for the induction of ERSE and upregulating proteins such as ER chaperones. It can also heterodimerize with XBP1s to upregulate ERAD components (201).

Temporally, ATF6 was shown to be activated quickly in response to ER stress, while the IRE1 and PERK pathways appear to be slightly delayed (40). Although not required (34), ATF6 upregulates *XBP1* mRNA, leading to higher activity of IRE1α and its pathway (30). XBP1s and ATF6 have very similar effector functions, including upregulating genes involved in correcting folding, trafficking, and degradation of misfolded proteins (202). Also similar to XBP1s, when a BiP inhibitor called subtilase cytotoxin was used, NFκB activation was reduced, showing the interplay between the UPR and NFκB disorders (203). They share a common function in upregulating ER expansion, but ATF6 does it independently through upregulating phosphatidylcholine and phospholipid synthesis (204). When ATF6α KO cells are cultured in stress conditions, they similarly handle stress as WT cells, but they do not return to equilibrium as quickly, nor do they survive chronic stress, instead showing increased CHOP, apoptosis, and lowered ER chaperones (205). ATF6 has been shown to bind to other proteins, such as calnexin and protein disulfide isomerases, allowing for increases in protein folding and ER chaperone functionality (206, 207). When only the absence of ATF6α, once challenged with tunicamycin, mice began to show signs of liver dysfunction and steatosis, showing the importance of ATF6α in lipid metabolism (208). Hepatic gluconeogenesis was also found to be related to ATF6 via the negative regulation of CREB-regulated transcription coactivation 2 (CRTC2), an important regulator of the aforementioned pathway (209). Another group also showed that a single knockout of ATF6α can impair spermatogenesis via the *TSSK4* gene in mice (210).

ATF6 has relations to Wolfram syndrome and cancer. Mutations in *WFS1* lead to the protein form transporting ATF6α to proteasomes for degradation, thus causing an aberrant UPR pathway in β cells (211). In regards to cancer, ATF6 was shown to promote proliferation and migration through MAPK signaling in cervical cancer (212). In colon cancer cells, ATF6 was found to activate mTOR, leading to sustained HSP90 expression and the prevention of apoptosis (213). Similarly, in colorectal cancer, GREM1, a protein involved in the epithelial-mesenchymal transition, was discovered to upregulate ATF6, resulting in metastasis promotion, linking this protein to the mTOR pathway (214). While therapeutically targeting ATF6 is not currently a large field, success has been seen in ischemia-reperfusion injuries (**Table 1**).

### 4.2 Alternative activation pathways of ATF6

ATF6 also has a non-canonical method of activation. During times of ER Ca^2+^ depletion, p90ATF6 can become partially glycosylated, ATF6(f), which reduces its binding with calreticulin, leading to increased export to the Golgi and under-glycosylated ATF6 becoming a sensing mechanism for ER stress (215). Sphingolipids, such as dihydrosphingosine and dihydroceramide, can also activate ATF6 in a non-canonical way, although they result in normal effector functions of ATF6 (216). In its inactive form in unstressed cells, ATF6 was shown to exist as monomers, dimers, and oligomers via disulfide bridges between the cysteine regions in the luminal domain, with only the monomeric version reaching the Golgi during stress (217). ERp18, an ER oxidoreductase, was shown to be a regulator of ATF6 release from the ER by associating with it and ensuring the monomeric form is released to the Golgi (218). To return to homeostasis, ATF6 can be negatively regulated by NUCB1, a Golgi-localized protein that is upregulated in response to ER stress (219). ATF4 has also been shown to have a regulatory role on ATF6; it can upregulate ATF6, as well as genes vital for the COPII vesicles, and when knocked out, it lessens ATF6 transport (220).

### 4.3 ATF6 pathway in CD8^+^ T cells

ATF6 is still relatively understudied for its impacts on CD8^+^ T cells. One group found that in a constitutive TNF-expressing model used for representing ileitis, ATF6, ATF4, and XBP1s can bind to the promoter regions of Granzyme B and BCL-2, an anti-apoptotic protein (221). In addition, using an *in vivo* CRISPR screen, ATF6 appears to be a restrictive element of T effector cell development, although further validation studies are needed (222). Similarly, a transcriptome study of tissue-resident memory chimeric antigen receptor T cells (CAR-T_RM_) uncovered that both ATF6 and ATF4 are upregulated, perhaps indicating that these two molecules may play a role in memory differentiation (223) (**Figure 2C**).

## 5 Conclusion

While recent work has highlighted the deeper intricacies of the UPR, more questions remain, particularly regarding the roles of each arm. For example, how does the UPR regulate the activation of each arm individually? There is also a lack of work regarding the exact function of ATF6. New technologies are consistently being refined that will allow for further exploration of these topics. As for CD8^+^ T cells, while there has been an effort to study the effects the UPR has on them, the exact mechanism by which they are regulated by the UPR is still unknown. The UPR is a broad system that encompasses three arms and several effector molecules. Research investigating the functionality of each molecule in each arm will be the goal, although this will take time. This leaves a broad space for researchers to investigate the UPR in CD8^+^ T cells due to several understudied aspects. For example, an outstanding question currently is what are the mechanisms that activate the UPR in CD8^+^ T cells. Some studies have uncovered those, but many are still needed to determine the exact effects of this activation and why it occurs. One study has shown in CD4^+^ T cells that XBP1 affects the abundance of glutamine transporters (136), although evidence in CD8^+^ T cells has been shown only by RNA-level changes (138). Further research is needed to validate the phenomenon and its effects on CD8^+^ T cells, albeit this evidence, as well as the evidence presented by the effects of PERK, indicate that metabolism, the UPR, and CD8^+^ T cells appear to be intricately linked. Work completed towards understanding the UPR has the potential to uncover novel ways to manipulate CD8^+^ T cells by using the UPR to increase their efficacy in the diseased setting.

## Author contributions

KN: Conceptualization, Writing – original draft, Writing – review & editing. BL: Conceptualization, Funding acquisition, Supervision, Writing – original draft, Writing – review & editing.
